# Genome Sequence of Foot-and-Mouth Disease Virus Serotype O Lineage Ind-2001d Collected in Vietnam in 2015

**DOI:** 10.1128/genomeA.00223-17

**Published:** 2017-05-04

**Authors:** Jonathan Arzt, Barbara Brito, Steven J. Pauszek, Ethan J. Hartwig, George R. Smoliga, Le T. Vu, Pham P. Vu, Carolina Stenfeldt, Luis L. Rodriguez, Ngo T. Long, Do H. Dung

**Affiliations:** a Foreign Animal Disease Research Unit, Plum Island Animal Disease Center, ARS, USDA, Orient Point, New York, USA; b Oak Ridge Institute for Science and Education, PIADC Research Participation Program, Oak Ridge, Tennessee, USA; c Regional Animal Health Office No. 6, Department of Animal Health, Ministry of Agriculture and Rural Development, Ho Chi Minh City, Vietnam; d Department of Animal Health, Ministry of Agriculture and Rural Development, Hanoi, Vietnam

## Abstract

In 2015, foot-and-mouth disease (FMD) virus lineage Ind-2001 was detected for the first time in Southeast Asia. This report contains the first near-complete genome sequence of a viral isolate from this lineage collected from an outbreak in Vietnam. This novel incursion has substantial implications for regional FMD control measures.

## GENOME ANNOUNCEMENT

Foot-and-mouth disease (FMD) is endemic in several countries in Asia and Africa (1, 2) and is considered one of the most important livestock diseases worldwide. FMD is caused by FMD virus (FMDV), a positive-sense single-stranded RNA aphthovirus from the *Picornaviridae* family (3). Currently, two serotypes of FMDV (A and O) are known to contribute to endemicity in mainland Southeast Asia. Serotype Asia1 was last confirmed in 2007 (2).

Ind-2001 is a distinct lineage of the FMDV serotype O, Middle East-South Asia (ME-SA) topotype. This lineage has been dominant in the Indian subcontinent since 2008 but is exotic to Southeast Asia (4). Limited outbreaks of this lineage were reported in the Middle East from 2008 to 2009 (http://www.wrlfmd.org/fmd_genotyping/2009/WRLFMD-2009-00005-United%20Arab%20Emirates-O.pdf). However, in 2013 to 2015, several outbreaks caused by sublineage Ind-2001d were reported from Libya, Morocco, Algeria, Tunisia, and the Persian Gulf States (5–8), and it is now endemic in some of these countries. In 2015, Ind-2001d was detected for the first time in Southeast Asia in cases reported from Vietnam and Laos (8).

The virus described herein, O/VIT/16451DLBPP04/2015, was isolated from an epithelial lesion collected from a pig in September 2015 in Ðắk Lắk Province, Vietnam. Acute and rapid onset of vesicular disease was detected in 45 of the 50 pigs present in the affected farm. Virus isolation was achieved by a single passage in BHK cells, as previously described (9). Viral RNA was extracted, and the partial 5′ untranslated region (UTR), the complete open reading frame (ORF), and the partial 3′ UTR were covered with three overlapping reverse transcription PCR (RT-PCR) amplicons and Sanger sequenced, as previously described (10). Chromatogram analysis and consensus sequence identification were performed with Sequencher version 5.4.6, as previously described (11).

Here, we report the near-complete sequence of the novel isolate FMDV O/VIT/16451DLBPP04/2015. This sequence codes for the complete 6,999-nucleotide (nt) ORF, 71 nucleotides in the 5′ UTR, and 38 nucleotides in the 3′ UTR. The ORF encodes a single polyprotein, which undergoes complex proteolytic processing, ultimately resulting in 4 structural proteins (VP1 to VP4) and 8 nonstructural proteins (lpro, 2A, 2B, 2C, 3A, 3B, 3C, and 3D). The highest identity with FMDV sequences publicly available is 98%, specifically from viruses collected from the Karnataka and Assam states in India between 2013 and 2014 (GenBank accession numbers KJ825804, KJ825809, KJ825805, KJ825808, KJ825806, KJ825807, KJ825803, and KJ825801). FMDV O/VIT/16451DLBPP04/2015 had no polyprotein indels with respect to the Indian sequences.

The outbreaks reported in Laos and Vietnam in 2015 were the first incursions of FMDV lineage Ind-2001 ever reported, to our knowledge, in Southeast Asia. Characterization of this virus has substantial implication for control measures, which are normally tailored toward endemic viruses and must be reassessed for new viruses. The regional situation with abundant susceptible hosts and a substantial diversity of cocirculating FMDV serotypes and lineages elevates the concern of the potential emergence of new viruses. Subsequent outbreaks, caused by independent introductions of the same sublineage in Myanmar, Thailand, and Vietnam in 2015 and 2016 (http://www.wrlfmd.org/fmd_genotyping/asia.html) further emphasize the importance of vigilance and characterization of novel introductions as they occur.

### Accession number(s).

The genome nucleotide sequence of O/VIT/16451DLBPP04/2015 described herein has been deposited in GenBank under the accession no. KY657269. The version described in this paper is the first version, KY657269.1.

## References

[1] World Organisation for Animal Health (OIE) 2009 Foot and mouth disease. World Organisation for Animal Health, Paris, France http://www.oie.int/fileadmin/Home/eng/Animal_Health_in_the_World/docs/pdf/Disease_cards/FOOT_AND_MOUTH_DISEASE.pdf.

[2] Brito BP, Rodriguez LL, Hammond JM, Pinto J, Perez AM 2017 Review of the global distribution of foot-and-mouth disease virus from 2007 to 2014. Transbound Emerg Dis 64:316–332. doi:10.1111/tbed.12373. 25996568

[3] Belsham GJ 2005 Translation and replication of FMDV RNA. Curr Top Microbiol Immunol 288:43–70. 15648174 10.1007/3-540-27109-0_3

[4] Subramaniam S, Mohapatra JK, Sharma GK, Biswal JK, Ranjan R, Rout M, Das B, Dash BB, Sanyal A, Pattnaik B 2015 Evolutionary dynamics of foot-and-mouth disease virus O/ME-SA/Ind2001 lineage. Vet Microbiol 178:181–189. doi:10.1016/j.vetmic.2015.05.015. 26049591

[5] Knowles NJ, Bachanek-Bankowska K, Wadsworth J, Mioulet V, Valdazo-González B, Eldaghayes IM, Dayhum AS, Kammon AM, Sharif MA, Waight S, Shamia AM, Tenzin S, Wernery U, Grazioli S, Brocchi E, Subramaniam S, Pattnaik B, King DP 2016 Outbreaks of foot-and-mouth disease in Libya and Saudi Arabia during 2013 due to an exotic O/ME-SA/Ind-2001 lineage virus. Transbound Emerg Dis 63:e431–e435. doi:10.1111/tbed.12299. 25483996

[6] OIE/FAO World Reference Laboratory for Foot-and-Mouth Disease (WRLFMD) 2013 OIE/FAO food-and-mouth disease reference laboratory network: annual report 2013. OIE/FAO Reference Laboratory Network for Food-and-Mouth Disease, Pirbright, United Kingdom http://www.wrlfmd.org/ref_labs/ref_lab_reports/OIE-FAO%20FMD%20Ref%20Lab%20Network%20Report%202013.pdf.

[7] OIE/FAO World Reference Laboratory for Foot-and-Mouth Disease (WRLFMD) 2014 OIE/FAO food-and-mouth disease reference laboratory network: annual report 2014. OIE/FAO Reference Laboratory Network for Food-and-Mouth Disease, Pirbright, United Kingdom http://www.wrlfmd.org/ref_labs/ref_lab_reports/OIE-FAO%20FMD%20Ref%20Lab%20Network%20Report%202014.pdf.

[8] OIE/FAO World Reference Laboratory for Foot-and-Mouth Disease (WRLFMD) 2015 WRLFMD quarterly report October to December 2015: reference laboratory contract report, foot-and-mouth disease. OIE/FAO Reference Laboratory Network for Food-and-Mouth Disease, Pirbright, United Kingdom http://www.wrlfmd.org/ref_labs/ref_lab_reports/OIE-FAO%20FMD%20Ref%20Lab%20Report%20Oct-Dec%202015.pdf.

[9] Pacheco JM, Arzt J, Rodriguez LL 2010 Early events in the pathogenesis of foot-and-mouth disease in cattle after controlled aerosol exposure. Vet J 183:46–53. doi:10.1016/j.tvjl.2008.08.023. 18930417

[10] Ludi A, Ahmed Z, Pomeroy LW, Pauszek SJ, Smoliga GR, Moritz M, Dickmu S, Abdoulkadiri S, Arzt J, Garabed R, Rodriguez LL 2016 Serotype diversity of foot-and-mouth-disease virus in livestock without history of vaccination in the Far North region of Cameroon. Transbound Emerg Dis 63:e27–e38. doi:10.1111/tbed.12227. 24735162 PMC4499489

[11] Pacheco JM, Piccone ME, Rieder E, Pauszek SJ, Borca MV, Rodriguez LL 2010 Domain disruptions of individual 3B proteins of foot-and-mouth disease virus do not alter growth in cell culture or virulence in cattle. Virology 405:149–156. doi:10.1016/j.virol.2010.05.036. 20580394

